# Gluten-Free Diet Adherence Tools for Individuals with Celiac Disease: A Systematic Review and Meta-Analysis of Tools Compared to Laboratory Tests

**DOI:** 10.3390/nu16152428

**Published:** 2024-07-26

**Authors:** Camila dos Santos Ribeiro, Rosa Harumi Uenishi, Alessandra dos Santos Domingues, Eduardo Yoshio Nakano, Raquel Braz Assunção Botelho, António Raposo, Renata Puppin Zandonadi

**Affiliations:** 1 Department of Nutrition, University of Brasília, Brasília 70910-900, Brazil; rosa.uenishi@gmail.com (R.H.U.); raquelbotelho@unb.br (R.B.A.B.); 2 Brasilia University Hospital, University of Brasília, Brasília 70840-901, Brazil; alessandra_gastro@hotmail.com; 3 Department of Statistics, University of Brasília, Brasilia 70910-900, Brazil; nakano@unb.br; 4 CBIOS (Research Center for Biosciences and Health Technologies), Universidade Lusófona de Humanidades e Tecnologias, Campo Grande 376, 1749-024 Lisboa, Portugal

**Keywords:** gluten-free diet, celiac disease, treatment adherence, laboratory test

## Abstract

This systematic review aimed to find the tool that best predicts celiac individuals’ adherence to a gluten-free diet (GFD). The Transparent Reporting of Multivariable Prediction Models for Individual Prognosis or Diagnosis (TRIPOD-SRMA) guideline was used for the construction and collection of data from eight scientific databases (PubMed, EMBASE, LILACS, Web of Science, LIVIVO, SCOPUS, Google Scholar, and Proquest) on 16 November 2023. The inclusion criteria were studies involving individuals with celiac disease (CD) who were over 18 years old and on a GFD for at least six months, using a questionnaire to predict adherence to a GFD, and comparing it with laboratory tests (serological tests, gluten immunogenic peptide—GIP, or biopsy). Review articles, book chapters, and studies without sufficient data were excluded. The Checklist for Critical Appraisal and Data Extraction for Systematic Reviews of Prediction Modeling Studies (CHARMS) was used for data collection from the selected primary studies, and their risk of bias and quality was assessed using the Prediction Risk of Bias Assessment Tool (PROBAST). The association between the GFD adherence determined by the tool and laboratory test was assessed using the phi contingency coefficient. The studies included in this review used four different tools to evaluate GFD adherence: BIAGI score, Coeliac Dietary Adherence Test (CDAT), self-report questions, and interviews. The comparison method most often used was biopsy (n = 19; 59.3%), followed by serology (n = 14; 43.7%) and gluten immunogenic peptides (GIPs) (n = 4; 12.5%). There were no significant differences between the interview, self-report, and BIAGI tools used to evaluate GFD adherence. These tools were better associated with GFD adherence than the CDAT. Considering their cost, application time, and prediction capacity, the self-report and BIAGI were the preferred tools for evaluating GFD adherence.

## 1. Introduction

Celiac disease (CD) is a chronic autoimmune condition that affects the small intestine with villous atrophy, causing intestinal and extraintestinal symptoms, and is triggered by the ingestion of gluten in genetically predisposed individuals [1,2]. It can trigger severe symptoms of malabsorption and nutritional deficiencies, such as anemia, diarrhea, constipation, short stature, muscular atrophy, and dermatitis herpetiformis, among others [1,3,4,5]. It is estimated that CD affects between 0.7% and 1.4% of the world population and is predominant in females; however, it is considered a neglected and underdiagnosed condition [5,6,7].

A gluten-free diet (GFD) is the only current treatment for the disease [8,9,10]. It can reverse the damage caused to the intestinal mucosa, primarily reducing morbidity and improving the quality of life of individuals with CD. GFDs entail completely restricting the consumption of gluten, a protein complex in wheat, rye, and barley, and its derivatives. Given the widespread presence of gluten in confectionery, bakery, pasta, and other industrialized products, adherence to a GFD can become a critical challenge for people affected by CD [11].

Several factors are involved in GFD adherence, such as the level of education received, the patient’s own perception and self-efficacy regarding the diet, knowledge, the duration of the GFD, instruction from qualified professionals, social restrictions, and even food labeling. The main reasons for GFD transgression are social events and changes in the food consumption environment [12,13]. However, assessing GFD adherence in individuals with CD is still challenging for researchers and health professionals, and how to monitor patients with CD is not well defined [11].

The methods for assessing GFD adherence are diverse and may have advantages and disadvantages. Despite being essential for adult diagnoses and the gold standard for evaluating mucosal recovery, biopsy is an invasive and high-cost method for monitoring the disease [14]. It is believed that it is possible to use alternative and less invasive methods to assess GFD adherence, such as interviews conducted by qualified professionals, the use of questionnaires, serological tests, or screening for gluten-derived peptides (GIPs) in feces or urine [9,15,16]. The serological tests recommended for predicting GFD adherence are tTG antibodies (tissue anti-transglutaminase), EMA (anti endomysium), and anti-DGPs (anti-deamidated gliadin peptides) of the IgA and IgG classes. Their high levels indicate low adherence, but negative values may not confirm strict adherence to the GFD and may be inaccessible in practice due to the lack of testing in healthcare services, patients refusing to have blood samples collected, and the cost [1,11,17]. The measurement of GIPs in feces and urine is the most recently established method; therefore, it is not yet widely available. It is expensive and has been rejected by patients [11].

GFD adherence must be guided and evaluated by health professionals with experience in CD, especially dietitians, through dietary interviews, food diaries, and questionnaires [11]. Questionnaires are simple, quick, and easy instruments that can be applied in clinical practice. Some of them are validated and widely used in studies, with good reliability [11,18,19,20]. However, there is no study that recommends the best tool to assess adherence to the DSG or which tool best predicts the GFD adherence of CD individuals, which is why this work is essential for contributing to the scientific literature and monitoring people with CD.

GFD adherence is essential in preventing symptoms, improving the quality of life of individuals with CD, and reducing health costs related to this condition [14]. However, confirming GFD adherence via an unreliable method may pose a risk to individuals with CD in terms of their diet [10]. Therefore, looking for a reliable, low-cost, and less invasive tool can benefit CD individuals, the health professionals who monitor their treatment, and researchers in the field. It is necessary to explore the literature on this topic better, expose the criteria used to evaluate GFD adherence in CD individuals, and, consequently, contribute to improving the monitoring of the dietary treatments used in CD and the quality of life of CD individuals. In this sense, this systematic review aimed to evaluate the non-invasive method that best predicts the adherence of individuals with celiac disease to a gluten-free diet.

## 2. Materials and Methods

### 2.1. Study Design

This systematic prediction review used the Transparent Reporting of Multivariable Prediction Models for Individual Prognosis or Diagnosis (TRI-POD-SRMA) guidelines for its construction. This type of review seeks to gather and summarize studies to predict health outcomes and inform prognoses or diagnoses [21]. The review was registered on the systematic review registration platform PROSPERO (International Prospective Register of Systematic Reviews) and is being analyzed by it under opinion number CRD42024518034.

The first stages consisted of general research on the topic, a search for previous systematic reviews, and the study feasibility study. The search question was “In adults with celiac disease undergoing treatment (gluten-free diet) for more than six months, which tool best predicts treatment adherence, compared to laboratory tests?”. A preliminary search strategy was carried out using the main keywords, following the acronym PICOT (P: person; I: intervention; C: comparison; O: outcome/result; and T: time), which is essential to guide the viability of a systematic review. A definitive search strategy was developed for each database, as well as the terms Mesh, DeCS, and Emtree (Table S1).

### 2.2. Eligibility Criteria

The following were included: (i) studies on adults older than 18 years old with a CD diagnosis and (ii) who have been undergoing treatment with a GFD for at least six months and (iii) studies which used questionnaires to predict adherence to the diet and compared it with a direct assessment method (tTG, EMA, DGP, GIP, or biopsy). The exclusion criteria were (i) studies carried out on people under 18 years old (ii) who had been on a GFD for less than six months (iii) and had no diagnosis of CD; (iv) review articles, book chapters, and conference proceedings; (v) studies without sufficient data for extraction; (vi) studies that did not evaluate adherence to a GFD.

### 2.3. Search and Data Extraction Strategy

Reviewers 1 and 2 (R1 and R2) collected the primary studies simultaneously and independently from eight scientific databases: PubMed, EMBASE, LILACS, Web of Science, LIVIVO, SCOPUS, Google Scholar, and Proquest. The search used the appropriate terms for each database (Table S1) without language or publication time restrictions.

### 2.4. Reference and Selection Manager

EndNote Web software was used to organize and remove 100% identical duplicates automatically. Then, the selected studies were exported to Rayyan software to organize the data and remove duplicates manually, before Phase 1 selection. The steps of organizing the data and duplicate removal were performed only by R1.

Two independent reviewers (R1 and R2) selected the articles to be included in two phases. Phase 1 selection involved independently reading the studies’ titles and abstracts in Rayyan software and applying the eligibility criteria. After that, differences were discussed and judged. Phase 2 selection consisted of the complete reading of the articles selected in Phase 1 and an additional search within the reference lists of the articles read in full to find studies with potential eligibility for this review. If disagreements arose in either phase, a third reviewer (R3) evaluated them before making a final decision. During Phase 2 selection, the exclusion criteria were numbered in order of importance, and a numbered reason was assigned to each excluded study.

### 2.5. Data Collection and Risk of Bias Analysis

To collect data from the primary studies that were included in Phase 2 selection within this study, the CHARMS checklist (CHecklist for critical Appraisal and data extraction for systematic Reviews of prediction Modeling Studies) was used [22]. Missing studies were asked for directly by email to the authors, with a maximum of three attempts made. Data collection was also conducted independently by R1 and R2. After data collection, the two reviewers (R1 and R2) completed the PROBAST list (Prediction Risk Of Bias Assessment Tool), also independently [23].

To extract data and generate tables and graphs, a Microsoft Excel^®^ (Office 365 version) model was independently used by the two reviewers and, at the end, a consensus meeting was held. R3 was consulted to solve divergencies. The file to be completed consisted of two spreadsheets, a template CHARMS and PROBAST, developed in previous studies [24].

### 2.6. Statistical Analysis and Meta-Analysis

The association between the GFD adherence calculated by the tool and laboratory test was assessed using the phi contingency coefficient. The phi coefficient measures the association between two binary variables and takes values between −1 and 1, with phi < 0 indicating a negative association, phi > 0 a positive association, and phi = 0 indicating no association. A meta-analysis of the studies that addressed the association between the GFD adherence calculated by the tool and laboratory tests was performed. Phi’s meta-analytic measurement (grouped value) was obtained using a random effects model.

Point estimates of the grouped phi values and their respective 95% confidence intervals (95% CI) are presented. The estimates were obtained by considering a single grouping of all the studies and also by considering subgroups according to the instrument adopted. The association between the GFD adherence calculated by the tool and laboratory test was considered significant (at a significance level of 5%) when the 95% CI did not contain a zero value. Additionally, the associations between two subgroups were considered significantly different when their respective CIs did not intersect. The analyses were performed using the R program’s metafor package, version 4.4.0 [25].

## 3. Results

### 3.1. Study Selection

**Figure 1:**
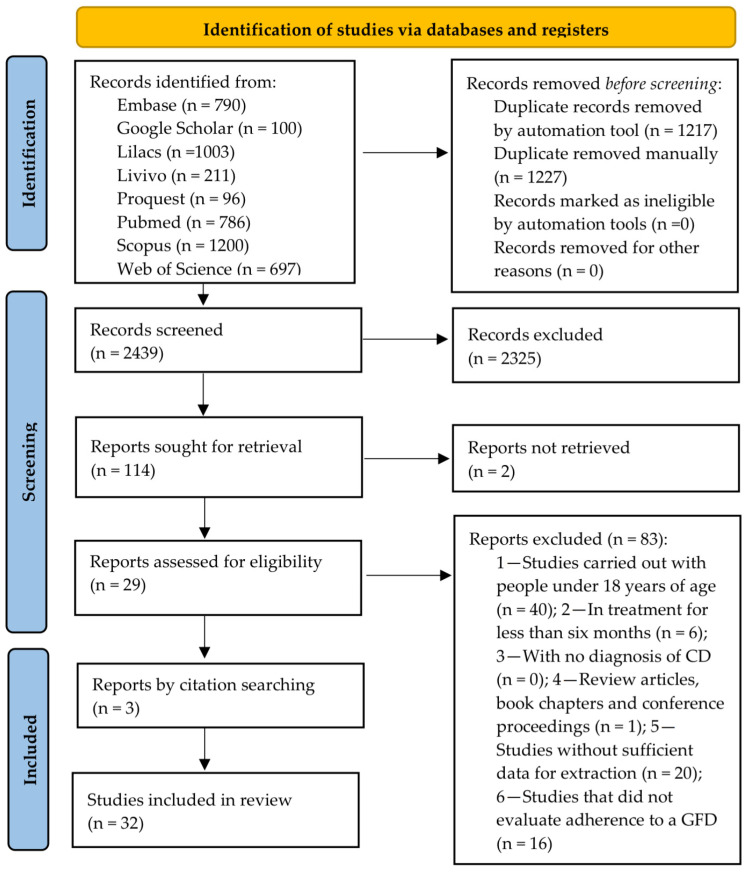
PRISMA flow diagram of literature search and selection criteria.

The database search resulted in 4883 articles, of which 2444 were duplicates. After Phase 1 selection, 2439 articles remained for the reading of their titles and abstracts, 114 of which were read in full and had their bibliographic references consulted (Phase 2 selection). The excluded studies and the reasons for their exclusion are presented in Table S2. Finally, 32 studies were eligible for this systematic review and 31 for a meta-analysis, as shown in the PRISMA flowchart (Figure 1).

### 3.2. The Studies’ Characteristics

The studies were performed from 1997 to 2024 and ranged from 18 to 694 (137.82 ± 145.24) participants. Thirty-one studies were characterized as cohort studies, and one was a randomized clinical trial study (Table 1). Most studies were performed in Italy [9,18,26,27,28,29,30,31,32,33,34,35] (n = 13; 40.6%), followed by Finland [36,37,38,39] (n = 4; 12.5%), the United Kingdom [35,40,41], and the United States [12,35,42] (n = 3, 9.3%), Argentina [43,44], Australia [45,46], Canada [47,48], Norway [49,50] (n = 2, 6.25%), while Paraguay, Poland, Romania, Spain, and Türkiye had one study each [20,35,51,52,53].

The studies included in this review used four different tools to evaluate GFDs: BIAGI scores [26], the Coeliac Dietary Adherence Test (CDAT) [19], self-report questionnaires, and interviews. Most of them used a biopsy [9,18,20,26,27,29,32,34,35,36,37,38,40,41,42,45,47,49,52] (n = 19; 59.3%), followed by serology [12,18,26,27,28,30,31,33,36,39,43,44,51,53] (n = 14; 43.7%) and GIPs [9,46,48,50] (n = 4; 12.5%). Of the 32 studies, most (n = 45; 46.8%) used the self-report method to evaluate GFD adherence [27,30,32,34,36,38,39,41,43,44,45,47,51,52,53], followed by the CDAT [9,12,20,40,46,48,49,50] (n = 8; 25%), BIAGI [18,26,31,33,40] (n = 5; 15.6%), and interviews [28,29,37,42] (n = 4; 12.5%), while only one used the Standardized Dietitian Evaluation (SDE) [20] and one of the studies used both the BIAGI and CDAT tools [35].

### 3.3. Meta-Analysis

**Figure 2:**
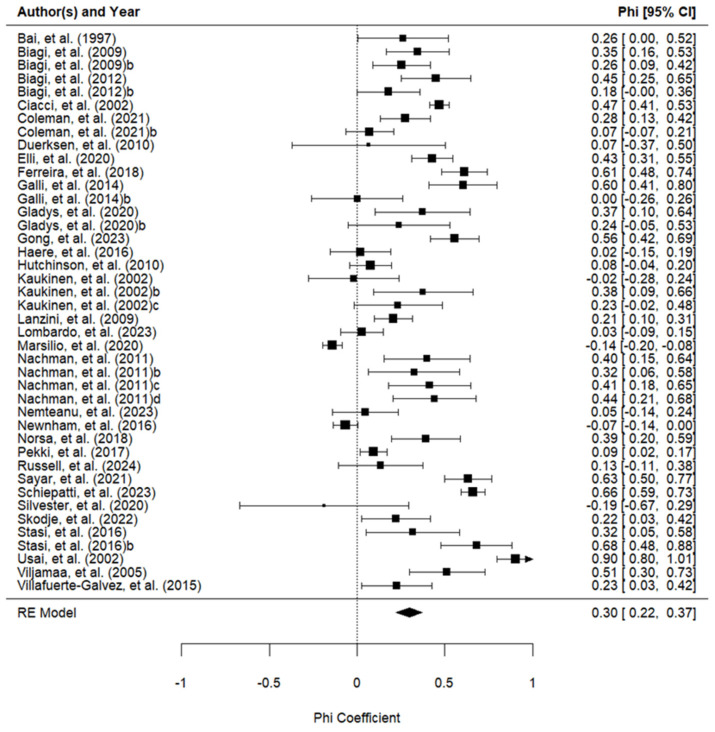
Forest plot of the phi coefficients of the association between the adherence measured with the tools and the adherence measured with the laboratory tests (42 studies) [9,12,18,20,26,27,28,29,30,31,32,33,34,35,36,38,39,40,41,42,43,44,45,46,47,48,49,50,51,53].

The results of the meta-analysis of the association between the GFD adherence calculated by the tool and laboratory tests are shown in Table 2 and Figure 2.

One study was excluded from the subgroup analysis because it used two instruments simultaneously (CDAT and BIAGI) and it was impossible to separate the data [35]. There were no significant differences between the interview, self-report, and BIAGI tools used to evaluate GFD adherence. These tools were better associated with GFD adherence than the CDAT. The Standardized Assessment of Dietitians (SDE) did not demonstrate an association with adherence to a GFD. However, it was evaluated in only one study and did not show statistically significant differences from any other instrument.

### 3.4. Risk of Bias and Concern

**Figure 3:**
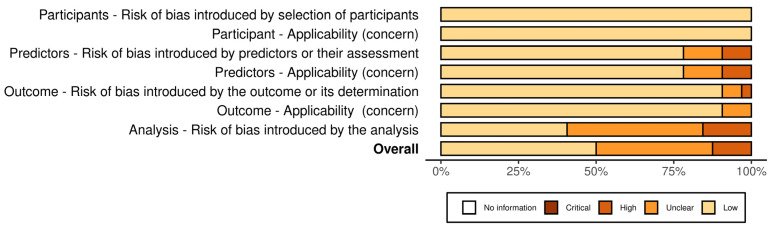
Risk of bias and concern in the included studies by domain, classified according to PROBAST.

**Figure 4:**
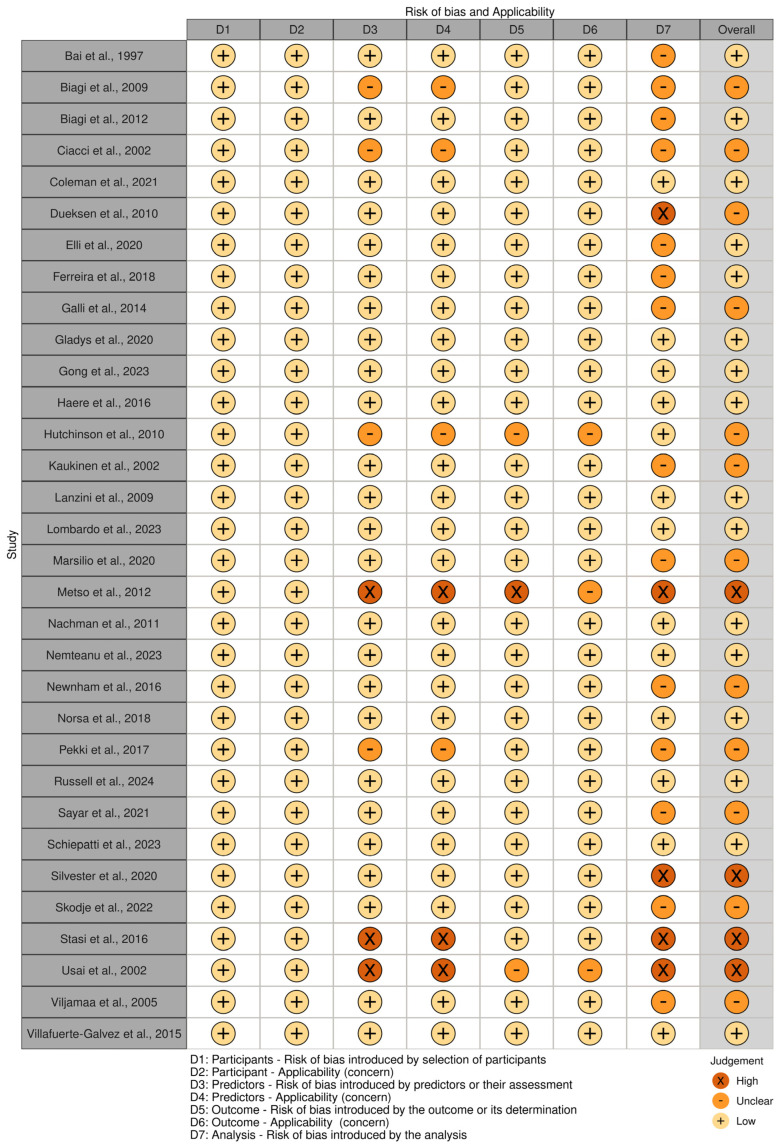
Risk of bias and applicability of the included studies, classified according to PROBAST [9,12,18,20,26,27,28,29,30,31,32,33,34,35,36,37,38,39,40,41,42,43,44,45,46,47,48,49,50,51,52,53].

Figure 3 and Figure 4 present the analysis of the risk of bias and concern in the included studies, classified according to PROBAST [23]. In total, 50% (n = 16) of the included studies demonstrated a low risk of bias [9,12,18,20,30,32,34,40,43,44,46,49,51,52,54]. A high risk of bias was identified in four studies [27,28,37,48], one of which used the self-report method [27], another an interview [28], and another the CDAT [48].

## 4. Discussion

This is the first systematic review with a meta-analysis to evaluate which non-invasive method best predicts the gluten-free diet adherence of individuals with celiac disease. Most studies were performed (n = 25; 78%) in Europe, and mainly in Italy (n = 12; 37.5%). Even though CD is considered a major worldwide public health problem and its prevalence varies by sex, age, and geographic location, the global estimates show that most of the population with CD is found in European countries [5,6], which justifies the large number of studies in Europe. In addition, about twenty years ago, Italy was considered the birthland of CD epidemiology due to the serological screening of its population; therefore, several studies on CD have been performed in this country [55,56].

Four methods were used in the studies and compared to laboratory tests: the CDAT, BIAGI, self-reports, and interviews. The self-report method was the most used tool to evaluate GFD adherence [27,30,32,34,36,38,39,41,43,44,45,47,51,52,53]. This method is characterized by an individual reporting whether or not they adhere to a GFD, in a dichotomous way (yes or no) or using a Likert scale (from never to always), or reporting their food intake through three-, four-, or seven-day food records to be analyzed or a dietary history. The dichotomous and Likert-scale methods used to evaluate GFD adherence are related to perceived adherence to the GFD, and their advantages are accessibility, quickness, and simplicity. However, records or dietary histories may take more time and be more complex, despite being helpful in evaluating food quality [57]. Although a food diary with a dietary interview was indicated by a study to adequately assess GFD adherence [11], the lack of classification standardization, the need for an expert, and memory bias can become barriers in practice. Self-reported adherence was positively correlated with dietitian assessments but not with the CDAT, according to authors [58]. However, some authors consider a self-report method for assessing GFD adherence problematic, since individuals with CD can incorrectly report (intentionally or not) their level of GFD adherence, leading to an over- or underestimation of their adherence to a GFD [59]. A prospective comparative study comparing the predictive value of self-reported GFD adherence to serological tests and expert dietitian evaluations showed that self-reporting is less reliable than serological tests, biopsies, and dietitian evaluations [60]. Despite this, our systematic review showed that self-reported GFD adherence did not differ from the BIAGI score and interviews and presented better accuracy than the CDAT tool. A structured interview conducted by a qualified professional can be a sensitive method for assessing GFD adherence, either through an SDE or through the self-reporting of diet by individuals with CD [1,10], as confirmed by our results. The SDE consists of a tool composed of structured questions, with food records lasting up to three days, assessing the patient’s ability to identify gluten in foods or other products, such as medicines and cosmetics. The disadvantages are that the SDE is subjective, takes more time, and a specialist is not always available in health services.

The BIAGI score was developed and validated in Italy in 2012 by a multicenter study [18,26]. Five studies included in this review used the BIAGI tool [18,26,31,33,40]. Four simple questions were developed based on the researchers’ clinical experience. One of the advantages is that the instrument can be applied even by those with no experience in CD and GFDs [18]. Studies have been using this tool with satisfactory reproducibility results [14,61,62]. Its classification varies from 0 to 4, with 0–1 points for those who do not follow a strict GFD; 2 points for those following a GFD but with important errors that require correction, and 3–4 points for those following a strict GFD. The authors state that it is possible to apply this to different ethnicities, with the last question (Do you only eat packaged foods guaranteed by the Celiac Association?) able to be omitted without affecting the final result in some countries, as local celiac societies may not provide lists of gluten-free packaged foods. Therefore, when validating the BIAGI tool in each country it will be applied as necessary.

The CDAT was created in 2009 in the USA from a meeting of specialists (gastro-enterologists, dietitians, psychologists, and CD individuals) to assess GFD adherence specifically [19]. After the meeting, they chose the five most important domains for evaluating GFD adherence: (1) symptoms related to CD, (2) specific knowledge of the disease, (3) self-efficacy, (4) reasons for maintaining a GFD, and (5) perceived adherence to the GFD. The CDAT consists of a seven-item questionnaire on a scale of 1 to 5. The minimum score is seven, and the maximum score is 35 points, with less than 13 points indicating good adherence [19]. This instrument has been translated into Spanish, Polish, and Norwegian [20,49,63,64], which are important for comparing different populations. However, its application takes time due to the number of items it contains and, in this systematic review, the CDAT presented the lowest association with laboratory tests.

The guidelines for celiac disease highlight that monitoring must be carried out through clinical evaluation, laboratory tests, and serology [1,65,66]. The normalization of laboratory tests indicates the remission of the disease, but the negativation of the tests is not immediate, and each test also has disadvantages that can limit its results. The quantification of antibodies, such as tTG, EMA, and DGP, is strongly recommended due to their high specificity and sensitivity [11,67]. Even though negative values cannot confirm a lack of exposure to gluten [11], it is evident that antibody values gradually decrease after months of a GFD [44]. Therefore, serology alone is not indicated to determine strict adherence to a gluten-free diet, and normalization does not indicate mucosal recovery [11,16].

Biopsy is considered the gold standard for evaluating mucosal healing; however, its invasive and high-cost nature means that the exam is not mandatory in monitoring CD, and the mucosal recovery time after a GFD is slow. Moreover, it varies for each individual. Studies differ on the indication period for biopsy, varying between repeating the biopsy after two years on a gluten-free diet or when symptoms and serological levels are altered [11,65,66,68]. In this systematic review, most of the studies performed a biopsy after a gluten-free diet was maintained for more than two years [20,29,38,40,41,42,45,47,49], which minimized the bias in the results.

Quantifying GIPs in feces and urine is a promising test that has also detected involuntary gluten consumption [46,69,70] and is recommended as a good direct approach to assessing adherence to a gluten-free diet and is helpful when available [1,11]; however, few studies used this comparator [9,46,48,50]. More studies are needed due to the individual variability in gluten metabolization and as their detectable time after ingestion is short (up to seven days) [11]. The consensus is that monitoring should be carried out frequently to assess the response to treatment and the adherence to a gluten-free diet [1,44,65,66]. Therefore, searching for less invasive, low-cost, and fast instruments to evaluate GFD adherence is essential.

This systematic review also has limitations. By including only studies on individuals over 18 years of age with celiac disease, many studies with the potential for analyzing the prediction of these instruments may have been excluded. Although biopsy is the gold standard for visualizing mucosal recovery, it can take up to five years for complete recovery in adults [10,17], which may have been a barrier in articles that used biopsy as a comparator over a short period for adherence assessments. In addition, the use of different methods (biopsy, serological, and GIP tests) may be a potential limitation, since the studies did not use the same method to evaluate GFD adherence. In order to minimize this, the tests were analyzed separately (Table 2).

A high risk of bias was only identified in four studies [27,28,37,48], and the concern was relatively low among the included studies. Accurately determining adherence to a GFD remains a challenge, particularly with respect to unintentional consumption. Both self-reports and tools rely on prior knowledge about the presence of gluten in foods, and this knowledge is not always accurate [59]. However, a standardized and straightforward tool facilitates the monitoring of individuals with celiac disease and guides professionals toward better management practices. Therefore, through this systematic review and meta-analysis, it is possible to emphasize the importance of using practical tools capable of predicting adherence to a GFD, thereby ensuring the effective monitoring of individuals with celiac disease.

## 5. Conclusions

There were no significant differences between the interview, self-report, and BIAGI tools used to evaluate GFD adherence. These tools were better associated with GFD adherence than the CDAT. Considering their cost, application time, potential accuracy of the level of GFD adherence, and prediction capacity, the self-report and BIAGI tools were considered the preferred tools to evaluate GFD adherence. These instruments are questionnaires completed by individuals. The evaluated tools depend on the CD patient’s responses in interviews or to questionnaires; therefore, it is necessary to raise awareness about the importance of accurately filling out these questionnaires and to expand patients’ knowledge about foods and the gluten-free diet to obtain the most accurate responses. Furthermore, additional studies are required to create standardized methods for evaluating diet adherence in various regions. These methods should be easily translatable and validated in multiple languages. They should also be simple to implement and highly accurate.

## Figures and Tables

**Table 1:** Baseline characteristics of the studies included.

Author, Year	Study Design	Enrolment Period	Country	n	Females (n)	Age	GFD Period (Month)	GFD Adherence Tool	%Adherence Using the Tool	Laboratory Test	%Adherence Using the Laboratory Test
Biagi et al., 2009 [26]	Cohort	NI	Italy	168	126	42.4 ± 13.9	82 (15–389)	BIAGI	79.7	Biopsy	91
				162	NI					EMA	70.3
Biagi et al., 2012 [18]	Cohort	2008–2011	Italy	141	108	34 ± 15	27 (6–298)	BIAGI	82.2	Biopsy	85.8
										EMA	73
Galli et al., 2014 [31]	Cohort	2009–2012	Italy	65	47	38 (18–70)	12	BIAGI	81.5	Biopsy	67.6
				57	NI					EMA/tTG	70
Marsilio et al., 2020 [33]	Cohort	2020	Italy	100	86	39.73 ± 13.51	79.68 ± 76.68	BIAGI	90	tTG	85
Coleman et al., 2021 [40]	Cohort	2013–2019	UK	201	136	50.3	>30	BIAGI	91	Biopsy	68.6
Villafuerte-Galvez et al., 2015 [12]	Cohort	2011–2012	USA	118	NI	53.6 ±1 15.4	118.8 ± 76.8	CDAT	73.7	tTG	82
Haere et al., 2016 [49]	Cohort	NI	Norway	127	79	55 ± 14	111.6 ± 60	CDAT	46.4	Biopsy	94.4
Gladys et al., 2020 [20]	Cohort	2015–2018	Poland	44	38	40.8	78 ± 86.4	CDAT	47.7	Biopsy	56.8
Silvester et al., 2020 [48]	Cohort	NI	Canada	18	12	41 (21–77)	24	CDAT	77.7	uGIPs fGIPs	33.3
Coleman et al., 2021 [40]	Cohort	2013–2019	England	201	136	50.3	>30	CDAT	49.7	Biopsy	68.6
Skodje et al., 2022 [50]	Cohort	NI	Norway	70	59	45	12	CDAT	53	fGIPs	91.4
Lombardo et al., 2023 [9]	Cohort	2019–2020	Italy	280	232	42.9	133.2 ± 122.4	CDAT	69.2	uGIPs	88.5
Russell et al., 2024 [46]	RCT	2020–2021	Australia	51	36	55 (44–62)	120 (60–168)	CDAT	72.5	fGIPs	23.5
Schiepatti et al., 2023 [35]	Cohort	2020–2022	Italy, Spain, UK, USA	694	491	>18	32 (15–61)	CDAT/BIAGI	83.5	Biopsy	77.3
Ciacci et al., 2002 [29]	Cohort	2002	Italy	390	299	27.9 ± 10.9	82.8 ± 90	Interview	42.5	Biopsy	76
Usai et al., 2002 [28]	Cohort	2002	Italy	66	66	46 (18–74)	>24	Interview	59	EMA/AGA	57.5
Metso et al., 2012 [37]	Cohort	2003–2006	Finland	26	22	>45	>12 meses	Interview	92.3	Biopsy	100
Gong et al., 2023 [42]	Cohort	2008–2019	USA	106	66	43.9	84	Interview	74.5	Biopsy	54.7
Gladys et al., 2020 [20]	Cohort	2020	Italy	44	38	40.8	78 ± 86.4	SDE	75	Biopsy	56.8
Bai et al., 1997 [43]	Cohort	1997	Argentina	22	NI	44 (21–73)	47 (23–75)	Self-reported	59	EMA/tTG	95.4
Kaukinen et al., 2002 [36]	Cohort	2002	Finland	57	NI	49 (22–73)	12 (12–216)	Self-reported	80.7	Biopsy	52.6
				87	63				87.3	EMA	87.3
				87	63				87.3	tTG	73.3
Viljamaa et al., 2005 [39]	Cohort	NI	Finland	97	51	51	144	Self-reported	83	tTG	91.7
Lanzini et al., 2009 [32]	Cohort	2009	Italy	465	356	31 (18–81)	16 (13–222)	Self-reported	85.8	Biopsy	79.5
Duerksen et al., 2010 [47]	Cohort	NI	Canada	21	19	50.5	116.4	Self-reported	71.4	Biopsy	71.4
Hutchinson et al., 2010 [41]	Cohort	2009	UK	234	202	>18	34.8	Self-reported	88	Biopsy	35
Nachman et al., 2011 [44]	Cohort	2004–2005	Argentina	53	48	18–66	12	Self-reported	60.3	TTG	62.2
							48		52.8	TTG	49
							12		60.3	tTG/DGP	79.2
							48		52.8	tTG/DGP	71.7
Newnham et al., 2016 [45]	Cohort	NI	Australia	44	NI	40 (18–71)	60	Self-reported	97.7	Biopsy	16
Stasi et al., 2016 [27]	Cohort	NI	Italy	39	NI	40	66 (13–261)	Self-reported	53.8	Biopsy	84.6
				52					86.5	EMA	75
Pekki et al., 2017 [38]	Cohort	NI	Finland	476	NI	55	96	Self-reported	98.7	Biopsy	58
Ferreira et al., 2018 [51]	Cohort	2015–2017	Paraguay	72	55	35.6 ± 12.4	294	Self-reported	68	tTG	44.4
Norsa et al., 2018 [34]	Cohort	2014–2015	Italy	63	NI	31.34	320 (1–432)	Self-reported	46	Biopsy	74.6
Elli et al., 2020 [30]	Cohort	2017–2018	Italy	197	159	44.6	87 ± 74	Self-reported	75.6	tTG	94.4
Sayar et al., 2021 [53]	Cohort	2010	Türkiye	78	68	36.8 ± 7.7	31	Self-reported	78.2	EMA/tTG	59
Nemteanu et al., 2023 [52]	Cohort	2016–2021	Romania	102	79	39.54 ± 12.70	22.6	Self-reported	27.4	tTG	71.5

Abbreviation: BIAGI = Biagi score; CDAT = Coeliac Dietary Adherence Test; GFD = gluten-free diet; SDE = Standardized Dietician Evaluation; AGA = gliadin antibody; tTG = tissue anti-transglutaminase antibody; EMA = anti-endomysium antibody; DGP = anti-deamidated gliadin peptide; fGIPs = gluten-derived peptides in feces; uGIPs = gluten-derived peptides in urine; RCT = randomized clinical trial; NI = no information.

**Table 2:** Meta-analysis results for the association between the GFD adherence calculated by tools and laboratory tests.

	Number of Studies	Grouped Estimation Phi (CI 95%)
TOTAL	42	0.297 (0.220; 0.372)
Tool used to evaluate GFD adherence *		
CDAT	8	0.112 (0.032; 0.192) ^A^
SDE	1	0.238 (−0.051; 0.528) ^AB^
BIAGI	8	0.242 (0.073; 0.410) ^AB^
Self-report	21	0.308 (0.209; 0.406) ^B^
Interview	3	0.641 (0.380; 0.903) ^B^
Laboratory test used to evaluate GFD adherence		
GIP	4	0.088 (−0.031; 0.207) ^A^
Biopsy	20	0.264 (0.163; 0.365) ^AB^
Serological (TTG, EMA, AGA)	18	0.378 (0.256; 0.501) ^B^
Tool X laboratory test *		
BIAGI and Serological	4	0.066 (−0.126; 0.258) ^A^
CDAT and GIP	4	0.088 (−0.031; 0.207) ^A^
Self-report and Biopsy	9	0.116 (0.016; 0.216) ^A^
CDAT and Biopsy	3	0.126 (−0.053; 0.304) ^AB^
CDAT and Serological	1	0.226 (0.027; 0.425) ^ABC^
SDE and Biopsy	1	0.238 (−0.051; 0.528) ^ABC^
BIAGI and Biopsy	4	0.410 (0.268; 0.551) ^BC^
Self-report and Serological	12	0.467 (0.384; 0.551) ^C^
Interview and Biopsy	2	0.489 (0.419; 0.559) ^C^
Interview and Serological	1	0.903 (0.796; 1.000) ^D^

* The study by Schiepatti et al. (2023) [35] adopted the CDAT/BIAGI questionnaires; therefore, it does not fit (in isolation) into either instrument. Groups with the same letters do not differ significantly. Abbreviations: BIAGI = Biagi score; CDAT = Coeliac Dietary Adherence Test; GFD = gluten-free diet; SDE = Standardized Dietician Evaluation; AGA = gliadin antibody; TTG = tissue anti-transglutaminase antibody; EMA = anti-endomysium antibody; GIP = gluten-derived peptide; CI = confidence interval.

## Supplementary Materials

The following supporting information can be downloaded at https://www.mdpi.com/article/10.3390/nu16152428/s1, Table S1: Database search strategy; Table S2: Excluded references and reasons.
