# Supplementary material for: Gluten-Free Diet Adherence Tools for Individuals with Celiac Disease: A Systematic Review and Meta-Analysis of Tools Compared to Laboratory Tests
Source: Nutrients. 2024 Jul 26;16(15):2428. doi: 10.3390/nu16152428 (PMC11314153; doi:10.3390/nu16152428)
Supplement: Supplementary file 1 [file nutrients-16-02428-s001.zip › nutrients-3094341-supplementary.pdf]

Table S1. Database search strategy.

PubMed<br>04/11/2023 08:01:15<br>Filter: All Fields	("celiac disease"[MeSH Terms] OR "celiac disease"[All Fields] OR "Gluten Enteropathy"[All Fields] OR "Gluten Enteropathies"[All Fields] OR "gluten sensitive enteropathy"[All Fields] OR "Gluten-Sensitive Enteropathies"[All Fields] OR "gluten sensitive enteropathy"[All Fields] OR "Gluten-Sensitive Enteropathies"[All Fields] OR "Celiac Sprue"[All Fields] OR "Nontropical Sprue"[All Fields] OR "Sprue"[All Fields] OR "Coeliac disease"[All Fields] OR "gluten-related disorders"[All Fields]) AND (((("diet, gluten free"[MeSH Terms] OR "Diet Therapy"[MeSH Terms:noexp] OR "Adherence"[All Fields] OR "Adherence Test"[All Fields]) AND "gluten-free diet"[All Fields]) AND "gluten-free diet"[All Fields]) OR "gluten free diets"[All Fields] OR "gluten free diets"[All Fields] OR "Compliance"[All Fields] OR "Diet Therapy"[All Fields] OR "dietary adherence"[All Fields] OR "effectiveness"[All Fields] OR "dietitian assessment"[All Fields]) AND ("Surveys and Questionnaires"[MeSH Terms] OR "Survey Methods"[All Fields] OR "Survey Method"[All Fields] OR "Survey Methodology"[All Fields] OR "Community Surveys"[All Fields] OR "Community Survey"[All Fields] OR "Community Survey"[All Fields] OR "Survey"[All Fields] OR "Questionnaire Design"[All Fields] OR "Questionnaire Designs"[All Fields] OR "Questionnaires"[All Fields] OR "Questionnaire"[All Fields] OR "validation study"[All Fields] OR "Validation Studies"[All Fields] OR "structured questionnaires"[All Fields] OR "survey studies"[All Fields])	786
LILACS<br>04/11/2023 08:16<br>Filter: title and abstract	("Celiac disease" OR "Gluten Enteropathy" OR "Gluten Enteropathies" OR "Gluten-Sensitive Enteropathy" OR "Gluten-Sensitive Enteropathies" OR "Gluten Sensitive Enteropathy" OR "Gluten-Sensitive Enteropathies" OR "Celiac Sprue" OR "Nontropical Sprue" OR "Sprue" OR "Coeliac disease" OR "gluten-related disorders" OR "doença celíaca" OR "enfermedad celíaca" OR "enteropatia glúten intuzida" OR "espru celíaco" OR "enteropatía por gluten") AND ("Diet therapy" OR "Adherence" OR "Adherence Test" OR "gluten-free diet" OR "gluten free diet" OR "Gluten-Free diets" OR "Gluten-Free Diets" OR "Compliance" OR "diet therapy" OR "dietary adherence" OR "effectiveness" OR "dietitian assessment" OR "dieta livre de glúten" OR " dieta sin gluten" OR "dieta livre de glúten") AND ("Surveys and Questionnaires" OR "Survey Methods" OR "Survey Method"	1002

	OR "Survey Methodology" OR "Community Surveys" OR "Community Survey" OR "Community Survey" OR "Survey" OR "Questionnaire Disign" OR "Questionnaire Designs" OR "Questionnaires" OR "Questionnaire" OR "validation study" OR "Validation Studies" OR "structured questionnaires" OR "survey studies" OR "inquéritos e questionários" OR "Encuestas y Cuestionarios" OR "cuestionario" OR "encuesta")	
SCOPUS<br>01/11/23	TITLE-ABS-KEY ("Celiac disease" OR "Gluten Enteropathy" OR "Gluten Enteropathies" OR "Gluten-Sensitive Enteropathy" OR "Gluten-Sensitive Enteropathies" OR "Gluten Sensitive Enteropathy" OR "Gluten-Sensitive Enteropathies" OR "Celiac Sprue" OR "Nontropical Sprue" OR "Sprue" OR "Coeliac disease" OR "gluten-related disorders" OR "doença celíaca" OR "enfermedad celíaca" OR "enteropatia glúten intuzida" OR "espru celíaco" OR "enteropatía por gluten") AND TITLE-ABS-KEY ("Diet therapy" OR "Adherence" OR "Adherence Test" OR "gluten-free diet" OR "gluten free diet" OR "Gluten-Free diets" OR "Gluten-Free Diets" OR "Compliance" OR "diet therapy" OR "dietary adherence" OR "effectiveness" OR "dietitian assessment" OR "dieta livre de glúten" OR " dieta sin gluten" OR "dieta livre de glúten") AND TITLE-ABS-KEY ("Surveys and Questionnaires" OR "Survey Methods" OR "Survey Method" OR "Survey Methodology" OR "Community Surveys" OR "Community Survey" OR "Community Survey" OR "Survey" OR "Questionnaire Disign" OR "Questionnaire Designs" OR "Questionnaires" OR "Questionnaire" OR "validation study" OR "Validation Studies" OR "structured questionnaires" OR "survey studies" OR "inquéritos e questionários" OR "Encuestas y Cuestionarios" OR "cuestionario" OR "encuesta")	1197
LIVIVO<br>01/11/23	(("Celiac disease" OR "Gluten Enteropathy" OR "Gluten Enteropathies" OR "Gluten-Sensitive Enteropathy" OR "Gluten-Sensitive Enteropathies" OR "Gluten Sensitive Enteropathy" OR "Gluten-Sensitive Enteropathies" OR "Celiac Sprue" OR "Nontropical Sprue" OR "Sprue" OR "Coeliac disease" OR "gluten-related disorders" OR "doença celíaca" OR "enfermedad celíaca" OR "enteropatia glúten intuzida" OR "espru celíaco" OR "enteropatía por gluten") AND ("Diet therapy" OR "Adherence" OR "Adherence Test" OR "gluten-free diet" OR "gluten free diet" OR "Gluten-Free diets" OR "Gluten-Free Diets" OR "Compliance" OR "diet therapy" OR "dietary adherence" OR "effectiveness" OR "dietitian assessment" OR "dieta livre de glúten" OR " dieta sin gluten"	210

	OR "dieta livre de glúten") AND ("Surveys and Questionnaires" OR "Survey Methods" OR "Survey Method" OR "Survey Methodology" OR "Community Surveys" OR "Community Survey" OR "Community Survey" OR "Survey" OR "Questionnaire Design" OR "Questionnaire Designs" OR "Questionnaires" OR "Questionnaire" OR "validation study" OR "Validation Studies" OR "structured questionnaires" OR "survey studies" OR "inquéritos e questionários" OR "Encuestas y Cuestionarios" OR "cuestionario" OR "encuesta"))	
EMBASE<br><br>01/11/23	#4. #1 AND #2 AND #3<br><br>#3. 'surveys and questionnaires'/de OR 'surveys and questionnaires' OR 'survey methods' OR 'survey method' OR 'survey methodology'/de OR 'survey methodology' OR 'community surveys'/de OR 'community surveys' OR 'community survey' OR 'survey'/de OR 'survey' OR 'questionnaire design'/de OR 'questionnaire design' OR 'questionnaire disign' OR 'questionnaire designs' OR 'questionnaires'/de OR 'questionnaires' OR 'questionnaire'/de OR 'questionnaire' OR 'validation study'/de OR 'validation study' OR 'validation studies'/de OR 'validation studies' OR 'structured questionnaires' OR 'survey studies' OR 'inquéritos e questionários' OR 'encuestas y cuestionarios' OR 'cuestionario' OR 'encuesta'<br><br>#2. 'adherence'/de OR 'adherence' OR 'adherence test' OR 'gluten-free diet'/de OR 'gluten-free diet' OR 'gluten free diet'/de OR 'gluten free diet' OR 'gluten-free diets' OR 'compliance'/de OR 'compliance' OR 'diet therapy'/de OR 'diet therapy' OR 'dietary adherence'/de OR 'dietary adherence' OR 'effectiveness' OR 'dietitian assessment' OR 'dieta sin gluten' OR 'dieta livre de glúten'<br><br>#1. 'celiac disease'/de OR 'celiac disease' OR 'gluten enteropathy'/de OR 'gluten enteropathy' OR 'gluten enteropathies' OR 'gluten-sensitive enteropathy'/de OR 'gluten-sensitive enteropathy' OR 'gluten sensitive enteropathy'/de OR 'gluten sensitive enteropathy' OR 'gluten-sensitive enteropathies' OR 'celiac sprue'/de OR 'celiac sprue' OR 'nontropical sprue'/de OR 'nontropical sprue' OR 'sprue'/de OR 'sprue' OR 'coeliac disease'/de OR 'coeliac disease' OR 'gluten-related disorders' OR 'doença celíaca' OR 'enfermedad celíaca' OR 'enteropatia glúten intuzida' OR 'espru celíaco' OR 'enteropatía por gluten'	1605

WEB OF SCIENCE<br><br>01/11/23	TS=("Celiac disease" OR "Gluten Enteropathy" OR "Gluten Enteropathies" OR "Gluten-Sensitive Enteropathy" OR "Gluten-Sensitive Enteropathies" OR "Gluten Sensitive Enteropathy" OR "Gluten-Sensitive Enteropathies" OR "Celiac Sprue" OR "Nontropical Sprue" OR "Sprue" OR "Coeliac disease" OR "gluten-related disorders" OR "doença celíaca" OR "enfermedad celíaca" OR "enteropatia glúten intuzida" OR "espru celíaco" OR "enteropatía por gluten") AND TS=("Diet therapy" OR "Adherence" OR "Adherence Test" OR "gluten-free diet" OR "gluten free diet" OR "Gluten-Free diets" OR "Gluten-Free Diets" OR "Compliance" OR "diet therapy" OR "dietary adherence" OR "effectiveness" OR "dietitian assessment" OR "dieta livre de glúten" OR " dieta sin gluten" OR "dieta livre de glúten") AND TS=("Surveys and Questionnaires" OR "Survey Methods" OR "Survey Method" OR "Survey Methodology" OR "Community Surveys" OR "Community Survey" OR "Community Survey" OR "Survey" OR "Questionnaire Design" OR "Questionnaire Designs" OR "Questionnaires" OR "Questionnaire" OR "validation study" OR "Validation Studies" OR "structured questionnaires" OR "survey studies" OR "inquéritos e questionários" OR "Encuestas y Cuestionarios" OR "cuestionario" OR "encuesta")	696
GOOGLE SCHOLAR<br><br>04/11/23<br><br>Filter: none	(("Celiac disease" OR "Gluten Enteropathy" OR "Gluten Enteropathies" OR "Celiac Sprue" OR "Coeliac disease") AND ("Diet therapy" OR "Adherence" OR "gluten-free diet" OR "Compliance" OR "dietary adherence") AND ("Surveys and Questionnaires" OR "Survey" OR "Questionnaire" OR "validation study" OR "structured questionnaires"))	100
PROQUEST<br><br>06/11/23<br><br>Limit to: full text<br><br>Source: todos<br><br>Document type: thesis<br><br>Language: all	("Celiac disease" OR "Gluten Enteropathy" OR "Coeliac disease") AND ("Diet therapy" OR "Adherence" OR "Compliance") AND ("Survey" OR "Questionnaire")	1896
BASE<br><br>06/11/23<br><br>Additional word forms	((Celiac disease) AND (Adherence) AND (Survey))	95

Table S2. Excluded references and reason.

Author	Reason
[1]	1
[2]	6
[3]	6
[4]	1
[5]	6
[6]	1
[7]	5
[8]	5
[9]	4
[10]	1
[11]	1
[12]	1
[13]	1
[14]	2
[15]	5
[16]	6
[17]	1
[18]	5
[19]	5
[20]	1
[21]	5
[22]	1
[23]	6
[24]	1
[25]	1
[26]	1
[27]	1
[28]	5
[29]	6
[30]	5
[31]	2
[32]	1
[33]	5
[34]	1
[35]	1
[36]	1
[37]	1
[38]	1

[39]	5
[40]	1
[41]	5
[42]	5
[43]	1
[44]	5
[45]	2
[46]	1
[47]	1
[48]	1
[49]	6
[50]	5
[51]	2
[52]	2
[53]	1
[54]	6
[55]	5
[56]	6
[57]	1
[58]	1
[59]	6
[60]	1
[61]	6
[62]	1
[63]	5
[64]	5
[65]	5
[66]	1
[67]	1
[68]	6
[69]	2
[70]	6
[71]	1
[72]	1
[73]	1
[74]	5
[75]	1
[76]	6
[77]	1
[78]	1
[79]	1
[80]	1
[81]	5
[82]	6
[83]	6

Reason for exclusion:

1) Studies carried out with people under 18 years of age;
2) In treatment for less than six months;

3) With no diagnosis of CD;
4) Review articles, book chapters and conference proceedings;
5) Studies without sufficient data for extraction;
6) Studies that did not evaluate adherence to a GFD

References:

1. Alharbi, I.S.; Sweid, A.M.; Memon, M.Y.; Alshieban, S.; Alanazi, A. Correlation of TTG IgA Level with Small Intestinal Histopathological Changes for Celiac Disease among Adult Saudi Patients. *J Transl Int Med* **2020**, *8*, 48–53, doi:10.2478/jtim-2020-0008.

2. Alhosain, A.I.; Alshammari, G.M.; Almoteri, B.L.; Mohammed, M.A.; Binobead, M.A.; Yahya, M.A. Long-Term Effect of Gluten-Free Diets on Nutritional Status, Body Composition, and Associated Factors in Adult Saudi Females with Celiac Disease. *Nutrients* **2022**, *14*, doi:10.3390/nu14102090.

3. Annibale, B.; Severi, C.; Chistolini, A.; Antonelli, G.; Lahner, E.; Marcheggiano, A.; Iannoni, C.; Monarca, B.; Delle Fave, G. *Efficacy of Gluten-Free Diet Alone on Recovery From Iron Deficiency Anemia in Adult Celiac Patients*; 2001;

4. Araya, M.; Mondragón, A.; Pérez-Bravo, F.; Roessler, J.L.; Alarcón, T.; Rios, G.; Bergenfreid, C. Celiac Disease in a Chilean Population Carrying Amerindian Traits. *J Pediatr Gastroenterol Nutr* **2000**, *31*, 381–386.

5. Ashorn, S.; Välineva, T.; Kaukinen, K.; Ashorn, M.; Braun, J.; Raukola, H.; Rantala, I.; Collin, P.; Mäki, M.; Luukkaala, T.; et al. Serological Responses to Microbial Antigens in Celiac Disease Patients during a Gluten-Free Diet. *J Clin Immunol* **2009**, *29*, 190–195, doi:10.1007/s10875-008-9255-7.

6. Atsawarungruangkit, A.; Silvester, J.A.; Weiten, D.; Green, K.L.; Wilkey, K.E.; Rigaux, L.N.; Bernstein, C.N.; Graff, L.A.; Walker, J.R.; Duerksen, D.R. Development of the Dietitian Integrated Evaluation Tool for Gluten-Free Diets (DIET-GFD). *Nutrition* **2020**, *78*, doi:10.1016/j.nut.2020.110819.

7. Barone, M.; Della Valle, N.; Rosania, R.; Facciorusso, A.; Trotta, A.; Cantatore, F.P.; Falco, S.; Pignatiello, S.; Viggiani, M.T.; Amoruso, A.; et al. A Comparison of the Nutritional Status between Adult Celiac Patients on a Long-Term, Strictly Gluten-Free Diet and Healthy Subjects. *Eur J Clin Nutr* **2016**, *70*, 23–27, doi:10.1038/ejcn.2015.114.

8. Bebb, J.R.; Lawson, A.; Knight, T.; Long, R.G. Long-Term Follow-up of Coeliac Disease - What Do Coeliac Patients Want? *Aliment Pharmacol Ther* **2006**, *23*, 827–831, doi:10.1111/j.1365-2036.2006.02824.x.

9. Bianchi, P.; Biagi, F.; Marchese, A.; Trotta, L.; Vattiato, C.; Brusco, G.; Corazza, G. P.42 THE GLUTEN-FREE DIET QUESTIONNAIRE: A PROSPECTIVE EVALUATION. *Digestive and Liver Disease* **2010**, *42*, S117–S118, doi:10.1016/s1590-8658(10)60156-9.

10. Bürgin-Wolff, A.; Dahlbom, I.; Hadziselimovic, F.; Petersson, C.J. *Antibodies Against Human Tissue Transglutaminase and Endomysium in Diagnosing and Monitoring Coeliac Disease*;

11. Butterworth, J.R.; Banfield, L.M.; Iqbal, T.H.; Cooper, B.T. Factors Relating to Compliance with a Gluten-Free Diet in Patients with Coeliac Disease: Comparison of White Caucasian and South Asian Patients. *Clinical Nutrition* **2004**, *23*, 1127–1134, doi:10.1016/j.clnu.2004.02.009.

12. Cadenhead, J.W.; Wolf, R.L.; Lebwohl, B.; Lee, A.R.; Zybert, P.; Reilly, N.R.; Schebendach, J.; Satherley, R.; Green, P.H.R. Diminished Quality of Life among Adolescents with Coeliac Disease Using Maladaptive Eating Behaviours to Manage a Gluten-Free Diet: A Cross-Sectional, Mixed-Methods Study. *Journal of Human Nutrition and Dietetics* **2019**, *32*, 311–320, doi:10.1111/jhn.12638.

13. Comino, I.; Real, A.; Vivas, S.; Síglez, M.Á.; Caminero, A.; Nistal, E.; Casqueiro, J.; Rodríguez-Herrera, A.; Cebolla, Á.; Sousa, C. Monitoring of Gluten-Free Diet Compliance in Celiac Patients by Assessment of Gliadin 33-Mer Equivalent Epitopes in Feces. *American Journal of Clinical Nutrition* **2012**, *95*, 670–677, doi:10.3945/ajcn.111.026708.

14. Cornell, H.J.; Czyzewska, A.; Macrae, F.A.; Rydzewska, G.; Nasierowska-Gutmejer, A.; Bednarczuk, A.; Stelmasiak, T. The Effect of Enzyme Supplementation on Symptoms and Duodenal Histology in Celiac Patients. *International Journal of Celiac Disease* **2016**, *4*, 40–47, doi:10.12691/ijcd-4-2-2.

15. Costas-Batlle, C.; Trott, N.; Jeanes, Y.; Seamark, L.; Gardiner, C. A Dietitian-Led Coeliac Service Helps to Identify and Reduce Involuntary Gluten Ingestion with Subsequent Reduction in the Frequency of Repeat Endoscopies. *Journal of Human Nutrition and Dietetics* **2023**, *36*, 1751–1759, doi:10.1111/jhn.13206.

16. De Chaisemartin, L.; Meatchi, T.; Malamut, G.; Fernani-Oukil, F.; Hosking, F.; Rault, D.; Bellery, F.; Cellier, C.; Dragon-Durey, M.A. Application of Deamidated Gliadin Antibodies in the Follow-up of Treated Celiac Disease. *PLoS One* **2015**, *10*, 1–16, doi:10.1371/journal.pone.0136745.

17. Deepak, C.; Berry, N.; Vaiphei, K.; Dhaka, N.; Sinha, S.K.; Kochhar, R. Quality of Life in Celiac Disease and the Effect of Gluten-Free Diet. *JGH Open* **2018**, *2*, 124–128, doi:10.1002/jgh3.12056.

18. Dewar, D.H. Celiac Disease: Management of Persistent Symptoms in Patients on a Gluten-Free Diet. *World J Gastroenterol* **2012**, *18*, 1348, doi:10.3748/wjg.v18.i12.1348.

19. Dimidi, E.; Kabir, B.; Singh, J.; Ageridou, A.; Foster, C.; Ciclitira, P.; Dubois, P.; Whelan, K. Predictors of Adherence to a Gluten-Free Diet in Celiac Disease: Do Knowledge, Attitudes, Experiences, Symptoms, and Quality of Life Play a Role? *Nutrition* **2021**, *90*, doi:10.1016/j.nut.2021.111249.

20. Dipper, C.R.; Maitra, S.; Thomas, R.; Lamb, C.A.; McLean-Tooke, A.P.C.; Ward, R.; Smith, D.; Spickett, G.; Mansfield, J.C. Anti-Tissue Transglutaminase Antibodies in the

Follow-up of Adult Coeliac Disease. *Aliment Pharmacol Ther* **2009**, *30*, 236–244, doi:10.1111/j.1365-2036.2009.04039.x.

21. Eigner, W.; Bashir, K.; Primas, C.; Kazemi-Shirazi, L.; Wrba, F.; Trauner, M.; Vogelsang, H. Dynamics of Occurrence of Refractory Coeliac Disease and Associated Complications over 25 Years. *Aliment Pharmacol Ther* **2017**, *45*, 364–372, doi:10.1111/apt.13867.

22. Fabiani, E.; Taccari, L.M.; R[auml]tsch, I.-M.; Di Giuseppe, S.; Coppa, G.V.; Catassi, C. Compliance with Gluten-Free Diet in Adolescents with Screening-Detected Celiac Disease: A 5-Year Follow-up Study. *Journal of Pediatrics* **2000**, *136*, 0841–0843, doi:10.1067/mpd.2000.105351.

23. Fang, H.; King, K.S.; Larson, J.J.; Snyder, M.R.; Wu, T.T.; Gandhi, M.J.; Murray, J.A. Undetectable Negative Tissue Transglutaminase IgA Antibodies Predict Mucosal Healing in Treated Coeliac Disease Patients. *Aliment Pharmacol Ther* **2017**, *46*, 681–687, doi:10.1111/apt.14250.

24. Farina, E.; Roncoroni, L.; Lombardo, V.; Scricciolo, A.; Vecchi, M.; Doneda, L.; Elli, L. Clinical Value of Tissue Transglutaminase Antibodies in Celiac Patients over a Long Term Follow-Up. *Nutrients* **2021**, *13*, doi:10.3390/nu13093057.

25. Fernández Miaja, M.; Suárez González, M.; Díaz Martín, J.J.; Jiménez Teviño, S.; Bousoño García, C.A. Analysis of Health-Related Quality Life in Celiac Patients. *Nutr Hosp* **2021**, *38*, 715–721, doi:10.20960/nh.03538.

26. Fernández Miaja, M.; José, J.; Martín, D.; Treviño, S.J.; Suárez González, M.; Bousoño García, C. *Study of Adherence to the Gluten-Free Diet in Coeliac Patients*; 2021; Vol. 94;.

27. Fueyo-Díaz, R.; Gascón-Santos, S.; Asensio-Martínez, Á.; Antonia Sánchez-Calavera, M.; Magallón-Botaya, R. *Transcultural Adaptation and Validation of the Celiac Dietary Adherence Test. A Simple Questionnaire to Measure Adherence to a Gluten-Free Diet*; 2016; Vol. 108;.

28. Galli, G.; Esposito, G.; Lahner, E.; Pilozzi, E.; Corleto, V.D.; Di Giulio, E.; Aloe Spiriti, M. a; Annibale, B. Histological Recovery and Gluten-Free Diet Adherence: A Prospective 1-Year Follow-up Study of Adult Patients with Coeliac Disease. *Aliment Pharmacol Ther* **2014**, *40*, 639–647, doi:10.1111/apt.12893.

29. Galli, G.; Amici, G.; Conti, L.; Lahner, E.; Annibale, B.; Carabotti, M. Sex–Gender Differences in Adult Coeliac Disease at Diagnosis and Gluten-Free-Diet Follow-Up. *Nutrients* **2022**, *14*, doi:10.3390/nu14153192.

30. Garzón-Benavides, M.; Ruiz-Carnicer, Á.; Segura, V.; Fombuena, B.; García-Fernandez, F.; Sobrino-Rodriguez, S.; Gómez-Izquierdo, L.; Montes-Cano, M.A.; Millan-Domínguez, R.; del Carmen Rico, M.; et al. Clinical Utility of Urinary Gluten Immunogenic Peptides in the Follow-up of Patients with Coeliac Disease. *Aliment Pharmacol Ther* **2023**, *57*, 993–1003, doi:10.1111/apt.17417.

31. George, J.B.E.; Leffler, D.A.; Dennis, M.D.; Franko, D.L.; Blom-Hoffman, J.; Kelly, C.P. *Psychological Correlates of Gluten-Free Diet Adherence in Adults With Celiac Disease*; 2009;

32. Gerasimidis, K.; Zafeiropoulou, K.; Mackinder, M.; Ijaz, U.Z.; Duncan, H.; Buchanan, E.; Cardigan, T.; Edwards, C.A.; McGrogan, P.; Russell, R.K. Comparison of Clinical Methods with the Faecal Gluten Immunogenic Peptide to Assess Gluten Intake in Coeliac Disease. *J Pediatr Gastroenterol Nutr* **2018**, *67*, 356–360, doi:10.1097/MPG.0000000000002062.

33. Gonzalez, A.F.; De La, M.; Temprano, P.; Inés, M.; Sanchez, P.; Sugai, E.; Longarini, G.I.; Costa, A.F.; Moreno, L.; Smecuol, E.; et al. *Validity and Reliability of the Celiac Disease Self-Management Inventory: A Self-Reported Tool to Assess Compliance of the Gluten-Free Diet in Adult Celiac Population Optimizing Behavioral Health Among Adolescents With Celiac Disease*;

34. Herman, M.L.; Rubio-Tapia, A.; Lahr, B.D.; Larson, J.J.; Van Dyke, C.T.; Murray, J.A. Patients With Celiac Disease Are Not Followed Up Adequately. *Clinical Gastroenterology and Hepatology* **2012**, *10*, 893-899.e1, doi:10.1016/j.cgh.2012.05.007.

35. Högberg, L.; Grodzinsky, E.; Stenhammar, L. Better Dietary Compliance in Patients with Coeliac Disease Diagnosed in Early Childhood. *Scand J Gastroenterol* **2003**, *38*, 751–754, doi:10.1080/00365520310003318.

36. Hollon, J.R.; Cureton, P. a; Martin, M.L.; Puppa, E.L.L.; Fasano, A. Trace Gluten Contamination May Play a Role in Mucosal and Clinical Recovery in a Subgroup of Diet-Adherent Non-Responsive Celiac Disease Patients. *BMC Gastroenterol* **2013**, *13*, 1–9, doi:10.1186/1471-230X-13-40.

37. Hopman, E.G.D.; Cessie, L.; Mary, §b; Von Blomberg, E.; Mearin, ¶m Luisa *Nutritional Management of the Gluten-Free Diet in Young People with Celiac Disease in The Netherlands*; Lippincott Williams & Wilkins, 2006; Vol. 43;.

38. Hopper, A.D.; Hadjivassiliou, M.; Hurlstone, D.P.; Lobo, A.J.; McAlindon, M.E.; Egner, W.; Wild, G.; Sanders, D.S. What Is the Role of Serologic Testing in Celiac Disease? A Prospective, Biopsy-Confirmed Study With Economic Analysis. *Clinical Gastroenterology and Hepatology* **2008**, *6*, 314–320, doi:10.1016/j.cgh.2007.12.008.

39. Hughey, J.J.; Ray, B.K.; Lee, A.R.; Voorhees, K.N.; Kelly, C.P.; Schuppan, D. Self-Reported Dietary Adherence, Disease-Specific Symptoms, and Quality of Life Are Associated with Healthcare Provider Follow-up in Celiac Disease. *BMC Gastroenterol* **2017**, *17*, doi:10.1186/s12876-017-0713-7.

40. Jivraj, A.; Hutchinson, J.M.; Ching, E.; Marwaha, A.; Verdu, E.F.; Armstrong, D.; Pinto-Sanchez, M.I. Micronutrient Deficiencies Are Frequent in Adult Patients with and without Celiac Disease on a Gluten-Free Diet, Regardless of Duration and Adherence to the Diet. *Nutrition* **2022**, *103–104*, doi:10.1016/j.nut.2022.111809.

41. Kabbani, T.A.; Goldberg, A.; Kelly, C.P.; Pallav, K.; Tariq, S.; Peer, A.; Hansen, J.; Dennis, M.; Leffler, D.A. Body Mass Index and the Risk of Obesity in Coeliac Disease Treated with the Gluten-Free Diet. *Aliment Pharmacol Ther* **2012**, *35*, 723–729, doi:10.1111/j.1365-2036.2012.05001.x.

42. Kivelä, L.; Popp, A.; Arvola, T.; Huhtala, H.; Kaukinen, K.; Kurppa, K. Long-Term Health and Treatment Outcomes in Adult Coeliac Disease Patients Diagnosed by Screening in Childhood. *United European Gastroenterol J* **2018**, *6*, 1022–1031, doi:10.1177/2050640618778386.

43. Koskinen, O.; Collin, P.; Lindfors, K.; Laurila, K.; Ma¨ki, M.; Kaukinen, K. *Usefulness of Small-Bowel Mucosal Transglutaminase-2 Specific Autoantibody Deposits in the Diagnosis and Follow-up of Celiac Disease*; 2010;

44. Koskinen, I.; Hervonen, K.; Huhtala, H.; Pasternack, C.; Salmi, T.; Reunala, T.; Collin, P.; Kaukinen, K. Mortality and Causes of Death in Different Celiac Disease Phenotypes during Long-Term Follow-Up. *Digestive and Liver Disease* **2022**, *54*, 1502–1507, doi:10.1016/j.dld.2022.04.016.

45. La Rosa Hernández, D.I.; Crespo Venega, M.I.; Del Valle Rivera, L.I.; Sánchez Castañeda, N.I.; Rosa Hernández, L.D.; Venegas, C.M.; Valle Rivera, D.L. *Cómo Citar Este Artículo*; 2016;

46. Laserna-Mendieta, E.J.; Casanova, M.J.; Arias, Á.; Arias-González, L.; Majano, P.; Mate, L.A.; Gordillo-Vélez, C.H.; Jiménez, M.; Angueira, T.; Tébar-Romero, E.; et al. Poor Sensitivity of Fecal Gluten Immunogenic Peptides and Serum Antibodies to Detect Duodenal Mucosal Damage in Celiac Disease Monitoring. *Nutrients* **2021**, *13*, 1–12, doi:10.3390/nu13010098.

47. Lau, M.S.; Mooney, P.D.; White, W.L.; Rees, M.A.; Wong, S.H.; Kurien, M.; Trott, N.; Leffler, D.A.; Hadjivassiliou, M.; Sanders, D.S. The Role of an IgA/IgG-Deamidated Gliadin Peptide Point-of-Care Test in Predicting Persistent Villous Atrophy in Patients With Celiac Disease on a Gluten-Free Diet. *American Journal of Gastroenterology* 2017, *112*, 1859–1867.

48. Laurikka, P.; Salmi, T.; Collin, P.; Huhtala, H.; Mäki, M.; Kaukinen, K.; Kurppa, K. Gastrointestinal Symptoms in Celiac Disease Patients on a Long-Term Gluten-Free Diet. *Nutrients* **2016**, *8*, doi:10.3390/nu8070429.

49. Lee, S.K.; Lo, W.; Memeo, L.; Rotterdam, H.; Green, P.H.R.; York, N. *Duodenal Histology in Patients with Celiac Disease after Treatment with a Gluten-Free Diet*; 2003;

50. Leffler, D.A.; Dennis, M.; Edwards George, J.B.; Jamma, S.; Magge, S.; Cook, E.F.; Schuppan, D.; Kelly, C.P. A Simple Validated Gluten-Free Diet Adherence Survey for Adults With Celiac Disease. *Clinical Gastroenterology and Hepatology* **2009**, *7*, doi:10.1016/j.cgh.2008.12.032.

51. Lichtwark, I.T.; Newnham, E.D.; Robinson, S.R.; Shepherd, S.J.; Hosking, P.; Gibson, P.R.; Yelland, G.W. Cognitive Impairment in Coeliac Disease Improves on a Gluten-Free Diet and Correlates with Histological and Serological Indices of Disease Severity. *Aliment Pharmacol Ther* **2014**, *40*, 160–170, doi:10.1111/apt.12809.

52. Lind, M. V.; Madsen, M.L.; Rumessen, J.J.; Vestergaard, H.; Gøbel, R.J.; Hansen, T.; Lauritzen, L.; Pedersen, O.B.; Kristensen, M.; Ross, A.B. Plasma Alkylresorcinols Reflect

Gluten Intake and Distinguish between Gluten-Rich and Gluten-Poor Diets in a Population at Risk of Metabolic Syndrome 1-3. *Journal of Nutrition* **2016**, *146*, 1991–1998, doi:10.3945/jn.116.236398.

53. Machado, J.; Gandolfi, L.; Coutinho De Almeida, F.; Malta Almeida, L.; Puppin Zandonadi, R.; Pratesi, R. Gluten-Free Dietary Compliance in Brazilian Celiac Patients: Questionnaire versus Serological Test. *Nutricion Clinica y Dietetica Hospitalaria* **2013**, *33*, 46–49, doi:10.12873/332gluten.

54. Mahadev, S.; Murray, J.A.; Wu, T.T.; Chandan, V.S.; Torbenson, M.S.; Kelly, C.P.; Maki, M.; Green, P.H.R.; Adelman, D.; Lebwohl, B. Factors Associated with Villus Atrophy in Symptomatic Coeliac Disease Patients on a Gluten-Free Diet. *Aliment Pharmacol Ther* **2017**, *45*, 1084–1093, doi:10.1111/apt.13988.

55. Maniero, D.; Lorenzon, G.; Marsilio, I.; D'Odorico, A.; Savarino, E.V.; Zingone, F. Assessment of Nutritional Status by Bioelectrical Impedance in Adult Patients with Celiac Disease: A Prospective Single-Center Study. *Nutrients* **2023**, *15*, doi:10.3390/nu15122686.

56. Midhagen, G. High Rate of Gastrointestinal Symptoms in Celiac Patients Living on a Gluten-Free Diet: Controlled Study. *Am J Gastroenterol* **2003**, *98*, 2023–2026, doi:10.1016/s0002-9270(03)00543-4.

57. Monzani, A.; Lionetti, E.; Felici, E.; Fransos, L.; Azzolina, D.; Rabbone, I.; Catassi, C. Adherence to the Gluten-Free Diet during the Lockdown for Covid-19 Pandemic: A Web-Based Survey of Italian Subjects with Celiac Disease. *Nutrients* **2020**, *12*, 1–12, doi:10.3390/nu12113467.

58. Moreno, M.D.L.; Cebolla, Á.; Munõz-Suano, A.; Carrillo-Carrion, C.; Comino, I.; Pizarro, Á.; León, F.; Rodríguez-Herrera, A.; Sousa, C. Detection of Gluten Immunogenic Peptides in the Urine of Patients with Coeliac Disease Reveals Transgressions in the Gluten-Free Diet and Incomplete Mucosal Healing. *Gut* **2017**, *66*, 250–257, doi:10.1136/gutjnl-2015-310148.

59. Nikniaz, Z.; Asghari Jafarabadi, M.; Ghaffarifar, S.; Saeedi, Z.; Akbari Namvar, Z.; Shirmohammadi, M. Psychometric Properties of the Persian Version of the Celiac Disease Adherence Test Questionnaire. *BMC Gastroenterol* **2020**, *20*, doi:10.1186/s12876-020-01396-8.

60. Nordyke, K.; Norström, F.; Lindholm, L.; Stenlund, H.; Rosén, A.; Ivarsson, A. Health-Related Quality of Life in Adolescents with Screening-Detected Celiac Disease, before and One Year after Diagnosis and Initiation of Gluten-Free Diet, a Prospective Nested Case-Referent Study. *BMC Public Health* **2013**, *13*, doi:10.1186/1471-2458-13-142.

61. O'Leary, C.; Wieneke, P.; Healy, M.; Cronin, C.; O'Regan, P.; Shanahan, F. Celiac Disease and the Transition from Childhood to Adulthood: A 28-Year Follow-Up. *American Journal of Gastroenterology* 2004, *99*, 2437–2441.

62. Olén, O.; Askling, J.; Ludvigsson, J.F.; Hildebrand, H.; Ekbom, A.; Smedby, K.E. Coeliac Disease Characteristics, Compliance to a Gluten Free Diet and Risk of Lymphoma by Subtype. *Digestive and Liver Disease* **2011**, *43*, 862–868, doi:10.1016/j.dld.2011.07.012.

63. Paarlahti, P.; Kurppa, K.; Ukkola, A.; Collin, P.; Huhtala, H.; Mäki, M.; Kaukinen, K. Predictors of Persistent Symptoms and Reduced Quality of Life in Treated Coeliac Disease Patients: A Large Cross-Sectional Study. *BMC Gastroenterol* **2013**, *13*, 75, doi:10.1186/1471-230X-13-75.

64. Paavola, A.; Kurppa, K.; Ukkola, A.; Collin, P.; Lähdeaho, M.L.; Huhtala, H.; Mäki, M.; Kaukinen, K. Gastrointestinal Symptoms and Quality of Life in Screen-Detected Celiac Disease. *Digestive and Liver Disease* **2012**, *44*, 814–818, doi:10.1016/j.dld.2012.04.019.

65. Paganizza, S.; Zanotti, R.; D'Odorico, A.; Scapolo, P.; Canova, C. Is Adherence to a Gluten-Free Diet by Adult Patients with Celiac Disease Influenced by Their Knowledge of the Gluten Content of Foods? *Gastroenterology Nursing* **2019**, *42*, 55–64, doi:10.1097/SGA.0000000000000368.

66. Pekki, H.; Kurppa, K.; Mäki, M.; Huhtala, H.; Sievänen, H.; Laurila, K.; Collin, P.; Kaukinen, K. Predictors and Significance of Incomplete Mucosal Recovery in Celiac Disease after 1 Year on a Gluten-Free Diet. *American Journal of Gastroenterology* **2015**, *110*, 1078–1085, doi:10.1038/ajg.2015.155.

67. Rodríguez-Herrera, A.; Reyes-Andrade, J.; Rubio-Escudero, C. Rationale for Timing of Follow-up Visits to Assess Gluten-Free Diet in Celiac Disease Patients Based on Data Mining. *Nutrients* **2021**, *13*, 1–8, doi:10.3390/nu13020357.

68. Roos, S.; Kärner, A.; Hallert, C. Psychological Well-Being of Adult Coeliac Patients Treated for 10 Years. *Digestive and Liver Disease* **2006**, *38*, 177–180, doi:10.1016/j.dld.2006.01.004.

69. Rubio-Tapia, A.; Rahim, M.W.; See, J.A.; Lahr, B.D.; Wu, T.T.; Murray, J.A. Mucosal Recovery and Mortality in Adults with Celiac Disease after Treatment with a Gluten-Free Diet. *American Journal of Gastroenterology* **2010**, *105*, 1412–1420, doi:10.1038/ajg.2010.10.

70. Sainsbury, K.; Mullan, B. Measuring Beliefs about Gluten Free Diet Adherence in Adult Coeliac Disease Using the Theory of Planned Behaviour. *Appetite* **2011**, *56*, 476–483, doi:10.1016/j.appet.2011.01.026.

71. Sainsbury, K.; Mullan, B.; Sharpe, L. A Randomized Controlled Trial of an Online Intervention to Improve Gluten-Free Diet Adherence in Celiac Disease. *American Journal of Gastroenterology* **2013**, *108*, 811–817, doi:10.1038/ajg.2013.47.

72. Sdepanian, V.L.; Batista De Morais, M.; Fagundes-Neto, U. *DOENÇA CELÍACA: Avaliação Da Obediência à Dieta Isenta de Glúten e Do Conhecimento Da Doença Pelos Pacientes Cadastrados Na Associação Dos Celíacos Do Brasil (ACELBRA)*;

73. Sharkey, L.M.; Corbett, G.; Currie, E.; Lee, J.; Sweeney, N.; Woodward, J.M. Optimising Delivery of Care in Coeliac Disease - Comparison of the Benefits of Repeat Biopsy and

Serological Follow-Up. *Aliment Pharmacol Ther* **2013**, *38*, 1278–1291, doi:10.1111/apt.12510.

74. Silvester, J.A.; Weiten, D.; Graff, L.A.; Walker, J.R.; Duerksen, D.R. Is It Gluten-Free? Relationship between Self-Reported Gluten-Free Diet Adherence and Knowledge of Gluten Content of Foods. *Nutrition* **2016**, *32*, 777–783, doi:10.1016/j.nut.2016.01.021.

75. Shepherd, S.J.; Gibson, P.R. Nutritional Inadequacies of the Gluten-Free Diet in Both Recently-Diagnosed and Long-Term Patients with Coeliac Disease. *Journal of Human Nutrition and Dietetics* **2013**, *26*, 349–358, doi:10.1111/jhn.12018.

76. Spatola, B.N.; Kaukinen, K.; Collin, P.; M??ki, M.; Kagnoff, M.F.; Daugherty, P.S. Persistence of Elevated Deamidated Gliadin Peptide Antibodies on a Gluten-Free Diet Indicates Nonresponsive Coeliac Disease. *Aliment Pharmacol Ther* **2014**, doi:10.1111/apt.12603.

77. Sugai, E.; Nachman, F.; Váquez, H.; González, A.; Andrenacci, P.; Czech, A.; Niveloni, S.; Mazure, R.; Smecuol, E.; Cabanne, A.; et al. Dynamics of Celiac Disease-Specific Serology after Initiation of a Gluten-Free Diet and Use in the Assessment of Compliance with Treatment. *Digestive and Liver Disease* **2010**, *42*, 352–358, doi:10.1016/j.dld.2009.07.011.

78. Tonutti, E.; Visentini, D.; Bizzaro, N.; Caradonna, M.; Cerni, L.; Villalta, D.; Tozzoli, R. *The Role of Antitissue Transglutaminase Assay for the Diagnosis and Monitoring of Coeliac Disease: A French-Italian Multicentre Study*; 2003; Vol. 56;.

79. Ukkola, A.; Mäki, M.; Kurppa, K.; Collin, P.; Huhtala, H.; Kekkonen, L.; Kaukinen, K. Changes in Body Mass Index on a Gluten-Free Diet in Coeliac Disease: A Nationwide Study. *Eur J Intern Med* **2012**, *23*, 384–388, doi:10.1016/j.ejim.2011.12.012.

80. Vahedi, K.; Mascart, F.; Mary, J.Y.; Laberenne, J.E.; Bouhnik, Y.; Morin, M.C.; Ocmant, A.; Velly, C.; Colombel, J.F.; Matuchansky, C. Reliability of Antitransglutaminase Antibodies as Predictors of Gluten-Free Diet Compliance in Adult Celiac Disease. *American Journal of Gastroenterology* **2003**, *98*, 1079–1087, doi:10.1111/j.1572-0241.2003.07284.x.

81. Valdimarsson, T.; Toss, G.; Löfman, O.; Ström, M. *Three Years' Follow-up of Bone Density in Adult Coeliac Disease: Significance of Secondary Hyperparathyroidism*;

82. Wang, N.; Truedsson, L.; Elvin, K.; Andersson, B.A.; R??nnelid, J.; Mincheva-Nilsson, L.; Lindkvist, A.; Ludvigsson, J.F.; Hammarstr??m, L.; Dahle, C. Serological Assessment for Celiac Disease in IgA Deficient Adults. *PLoS One* **2014**, doi:10.1371/journal.pone.0093180.

83. Zanini, B.; Marullo, M.; Villanacci, V.; Salemme, M.; Lanzarotto, F.; Ricci, C.; Lanzini, A. Persistent Intraepithelial Lymphocytosis in Celiac Patients Adhering to Gluten-Free Diet Is Not Abolished Despite a Gluten Contamination Elimination Diet. *Nutrients* **2016**, *8*, 525, doi:10.3390/nu8090525.
